# Socioeconomic Burden of Critically Ill Patients: A Descriptive Study

**DOI:** 10.7759/cureus.35598

**Published:** 2023-02-28

**Authors:** Mamta Gehlot, Satyapriya Mohanty, Mahalingam Venkateshan, B Gomathi, Asha Shetty, Prasanta K Das, Priyadarshini Mishra, Arvind Pandey, Debasish Das

**Affiliations:** 1 Nursing, All India Institute of Medical Sciences, Bhubaneswar, Bhubaneswar, IND; 2 Cardiothoracic Surgery, All India Institute of Medical Sciences, Bhubaneswar, Bhubaneswar, IND; 3 Nursing, Sum Nursing College, Siksha O Anusandhan (SOA) University, Bhubaneswar, IND; 4 Anesthesiology and Critical Care, All India Institute of Medical Sciences, Bhubaneswar, Bhubaneswar, IND; 5 Ophthalmology, All India Institute of Medical Sciences, Bhubaneswar, Bhubaneswar, IND; 6 Cardiothoracic Surgery, Institute of Medical Sciences, Banaras Hindu University, Varanasi, IND; 7 Cardiology, All India Institute of Medical Sciences, Bhubaneswar, Bhubaneswar, IND

**Keywords:** india, family, socio-economic burden, intensive care units, critically ill patients

## Abstract

Background

The cost of critical illness treatment is generally recognized as expensive and increasing in India. Critical illness of the individual will affect the socioeconomic status of the individual and the family. The direct and indirect costs of intensive care and its impact on the socioeconomic status of critically ill patients and their families need to be estimated. The present study was carried out to evaluate the socioeconomic burden of critically ill patients admitted to ICUs in Eastern India.

Methods

A descriptive survey was conducted to measure the socioeconomic burden. One hundred fifteen critically ill patients and their family members were conveniently selected for the study. Critically ill patients admitted to ICUs and those who were bedridden for more than seven days along with anyone the family member, i.e., spouse, father, or mother, were included in the study to estimate the impact of long-term illness on the care providers in the family. Socio-demographic and socioeconomic burdens were analyzed through the interview method.

Results

Half (49.6%) of the critically ill patients were heads of the family, and their employment is the primary source of income for the family members. Most (60.9%) of the patients belonged to lower socioeconomic status. Critically ill patients spend a maximum (38169.6±3996.2) amount for pharmaceutical expenses. Eventually, the family members accompanying patients lost maximum working days because of the long length of hospital stay. Below upper-lower (p=0.046) class socioeconomic family, age less than 40 (p=0.018) years, and those families depending (p=0.003) on patients' income significantly reported higher socioeconomic burden.

Conclusions

Critical care hospitalization of patients increases the socioeconomic burden on the whole family, especially in lower-middle-income countries like India. It soberly affects younger age group patients with low socioeconomic status and families depending on the patient's income during their man days.

## Introduction

India has one of the most highly privatized healthcare systems in the world [1]. Private hospitals and health care agencies contribute ninety percent of the ICU beds, and only 10% of ICU beds are available in public hospitals. At the same time, 40% of the population who need ICU care belongs below the poverty line (BPL). As a result, this mismatch contributing to a single-time admission to ICU may push the families into extreme poverty, and the consequences are catastrophic. As a result of the high payments through out-of-pocket (OOP) expenses, approximately 8% of the population remains BPL [2]. It is estimated that ICU admission costs maybe 100 times per capita income, and a single ICU admission can bring families to extreme poverty [3]. Data from a government-funded tertiary care hospital revealed that the mean OOP expenditures per patient bed-day in the ICU was Indian rupee (INR) 13,194 (US$168.55), and 51% of the patients' families depleted their lifetime saving to meet OOP expenditures. Interestingly, medicines, drugs, and OOP were the major costs of patients admitted to government-funded public hospitals [4]. It is also reported that the ICUs from low-income countries had withdrawn life-sustaining treatments from the patients based on the requests of patients' families to avoid costly bills. Unfortunately, these patients would have had reasonable chances of survival if treatment had continued [5].

The provision of cost-effective intensive care and reduction of OOP expenditure of families of patients admitted to ICUs pose a challenge in India. The major reason could be the lack of penetration of government-funded insurance schemes. Despite several government-funded health insurance schemes (GFHI) available in India, OOP expenditure is still high. This may lead to inequitable access to health care services by the poor. The penetration of GFHI was also skewed. Non-poor and urban take the lion's share as compared to their counterparts. It implies the failure of schemes to reach the poor, who spend a major share of their household income during hospitalization. The household survey (2017-2018) revealed a wide variation between claims of official government data and household surveys in terms of GFHI coverage in India [6]. Notably, the uptake of private insurance among middle and poor economies is almost non-existing. A multicentric study (120 ICUs) reported that only 17% of the ICU patients' costs were borne by their insurance or employer, and 81% of the patients made their payments from their pockets [7].

Need for the study

The lack of insurance, direct cost of ICU care, and indirect costs such as stay, travel, job loss, rehabilitation, and follow-up care may generate a significant socioeconomic burden on the family of a patient receiving ICU care. Although there was a lot of literature on the burden of caregivers and families of ICU survivors, the study examining the socioeconomic burden of patients' families admitted to public hospital ICUs was limited. In addition, the nature and characteristics of the burden of patients' families admitted to public hospitals ICUs are unique due to the cost of the procedure, diet, essential medication, investigations, sophisticated equipment, and life-sustaining treatments being borne by the government. However, long queues, uneven distribution of tertiary care facilities, accessibility issues, care continuity, delayed treatments, and indirect costs such as travel, stay, caregiver expenses, and loss of daily wages may contribute to a significant burden to the families. Hence, this study aimed to assess the socioeconomic burden of critically ill patients admitted to the tertiary care hospital in Bhubaneswar.

## Materials and methods

Study design

A descriptive survey was conducted, and convenience sampling was used to recruit the family members of critically ill patients.

Inclusion criteria

Critically ill patients bedridden for more than seven days in the ICU, aged more than 18 years, along with any family members, i.e., father, mother, or spouse, were included in the study. A total of 115 family members of critically ill patients were enrolled in this study.

Exclusion criteria

Patients aged under 18 and over 90 years and pregnant females were excluded from the study.

Study setting

The study was carried out in tertiary care teaching hospital in Odisha, an Indian state located in the Eastern part of the country. It is a 1,018 noncritical care and 109 critical care bedded hospital. Also, it has all the specialty and super specialty critical beds like medical ICU, surgical ICU, neuro medicine and neurosurgery ICU, cardiology and cardiothoracic ICU, pediatric ICU, respiratory ICU, gastro surgery ICU, transplant ICU, hemato-oncology ICU, trauma ICU, and steps down ICU. Ethical clearance was obtained from the Institutional Ethical Committee (IEC; Ref# IEC/AIIMS BBSR/Nursing/2021-22/14) and written informed consent was obtained from the study subjects.

Study tools and method of data collection

Investigator developed the tool and systematically tested its validity and reliability. The tool consists of four sections, section A includes the socio-demographic characteristics of the study participants, section B includes the economic status of the family, section C includes direct and indirect cost expenditure of the treatment, and section D includes the socioeconomic burden on the patient and family. After screening the study participants based on inclusion and exclusion criteria, informed consent was taken, and a 25-30-minute face-to-face interview was conducted based on the tool.

## Results

Socio-demographic data

Table 1 shows the demographic and socioeconomic variables of study participants. More than half (51% ) of the study participants were aged more than 51 years, 55.7% were male, and approximately three fourth (77.4%) of them were residing in rural areas. Every second (49%) of the patient is the head of the family, most (98.3%) of them were employed, and 61% were from lower socioeconomic class families.

**Table 1:** Distribution of demographic and socioeconomic variables of patients.

Variables	Classification	f	%
Age group	≤40	35	30.4
	41-50	21	18.3
	51-60	32	27.8
	>60	27	23.5
Gender	Male	64	55.7
	Female	51	44.3
Locality	Rural	89	77.4
	Urban	26	22.6
Occupation	Unemployed	50	43.5
	Elementary Occupation	2	1.7
	Plant & Machine Operators and Assemblers	2	1.7
	Craft & Related Trade Workers	1	0.9
	Skilled Agricultural & Fishery Workers	34	29.6
	Skilled Workers and Shop & Market Sales Workers	15	13
	Clerks	2	1.7
	Technicians and Associate Professionals	1	0.9
	Professionals	8	7
Education	No Formal Education	22	19.1
	Primary school certificate	18	15.7
	Middle school certificate	17	14.8
	High school certificate	37	32.2
	Intermediate or diploma	2	1.7
	Graduate	18	15.7
	Professional or Honours	1	0.9
Head of the family	Patient	57	49.6
	Father	13	11.3
	Son	16	13.9
	Husband	23	20
	Daughter	2	1.7
	Other family members	4	3.5
Occupation of the head of the family	Unemployed	2	1.7
	Elementary Occupation	43	37.4
	Plant & Machine Operators and Assemblers	7	6.1
	Craft & Related Trade Workers	7	6.1
	Skilled Agricultural & Fishery Workers	20	17.4
	Skilled Workers and Shop & Market Sales Workers	20	17.4
	Clerks	5	4.3
	Technicians and Associate Professionals	1	0.9
	Professionals	9	7.8
	Legislators, Senior Officials & Managers	1	0.9
Socioeconomic class	Upper (I)	1	0.9
	Upper Middle (II)	14	12.2
	Lower Middle (III)	30	26.1
	Upper Lower (IV)	66	57.4
	Lower (V)	4	3.5
Monthly income of the family	≤10,001	56	48.7
	10,002-29,972	40	34.8
	29,973-49,961	10	8.7
	49,962-74,755	1	0.9
	74,755-99,930	4	3.5
	99,931-199,861	3	2.6
	≥199,862	1	0.9

Table 2 shows the direct and indirect cost expenditure of the treatment. The direct cost of treatment has been divided into four components in this study, i.e., institutional cost, pharmacies cost, cost of transportation, and cost of food and stay. Since the study setting is a government aid institution, 86.1% of patients reported they did not spend any institutional medical cost, and 97.4% reported spending more than 10,000 INR for pharmacy and medication purchases. More than three-fourths (77.4%) of the patients spent more than 5000 INR for transportation, and 94.8% reported they have to spend more than 5000 INR for food and stay for family and patients. The indirect cost of the hospitalization has been estimated by calculating the loss of man-days. Half (49.6%) of the patients and three-fourths (76.5%) of the patient's relatives reported losing more than 14 days of income due to hospitalization. Also, one-fourth (25.2%) of patients informed that they had lost more than 14 days of their job because of a scheduled hospital visit. More than half (57.4%) of patient relatives stated that the critically ill patient treatment cost had been paid through government health insurance schemes, and 42.6% of the patients paid from their savings.

**Table 2:** Direct and indirect cost expenditure of the treatment.

Direct Cost		f	%
Medical Instituitons Cost	No expenses	99	86.1
	Below 5000 INR	12	10.4
	Above 5000 INR	4	3.5
	Mean±SD	6404.4±24765.1	
Pharmacy Cost	No Expenses	3	2.6
	≤10,000 INR	29	25.2
	10001-20000 INR	42	36.5
	20001-30000 INR	18	15.7
	>30000 INR	23	20
	Mean ± SD in INR	38169.6 ± 139968.2	
Transportation Cost	No Expenses	26	22.6
	≤5000 INR	52	45.2
	5001-10000 INR	26	22.6
	>10000 INR	11	9.6
	Mean ± SD in INR	4583.9 ± 5052.3	
Other Costs in Hospital	No Expenses	6	5.2
	≤5000 INR	47	40.9
	5001-10000 INR	30	26.1
	>10000 INR	32	27.8
	Mean ± SD in INR	10798.4 ± 15358.7	
Indirect Cost			
Loss of man-days by the patient during hospitalization	≤14 days	58	50.4
	14-28 days	26	22.6
	28-42 days	12	10.4
	>42 days	19	16.5
Loss of man-days by the patient due to visiting hospital before hospitalization	≤7 days	86	74.8
	7-14 days	7	6.1
	14-21 days	10	8.7
	>21 days	12	10.4
Loss of man-days by the patient’s relative due to patient's hospital treatment	≤14 days	27	23.5
	14-28 days	35	30.4
	28-42 days	19	16.5
	>42 days	34	29.6
Source of medical expenditure	Self-payment	49	42.6
	Below Poverty Line Scheme (BPL)	43	37.4
	Government Health Scheme (BSKY)	22	19.1
	Other Health Insurance	1	0.9

Socioeconomic burden on the patient and family members

Table 3 depicts the socioeconomic burden of critically ill patients on family and family members. Most of them reported that their family members lost their job and income because of the hospitalization of the patient (40%). Also, the loss of patient income has affected the family's financial status. Every tenth family (10.4%) reported leisure activities being stopped because of the patient's hospitalization. One-fourth (26.1%) of the family members stated that because of the hospitalization of the patient, few family members were psychologically affected and asked for help in treating these psychological symptoms.

**Table 3:** Socioeconomic burden of critically ill patients.

	Not Agree					Neither Agree Nor Disagree				Agree		
Financial Burden	f			%		f		%		f		%
Patient income loss in the family affected the family’s financial status.	56			48.7		13		11.3		46		40
A family member lost their job and income because of the hospitalization of the patient	49			42.6		18		15.7		48		41.7
Patient hospital expenditure affected the family’s financial status	67			58.3		33		28.7		15		13
Additional arrangements are taking extra expenditure?	75			65.2		25		21.7		15		13
The additional loan is taken because of all the savings spent on the patient?	70			60.9		23		20		22		19.1
Are recreational activities cut off because of patient admission?	62			53.9		33		28.7		20		17.4
Disturbance of routine family activities												
Patient occupation or study is interrupted?	59		51.3				7		6.1	49		42.6
Patient household work support is interrupted?	62		53.9				12		10.4	41		35.7
Have family members’ activities been disrupted?	44		38.3				36		31.3	35		30.4
Is patient behavior disrupting family activities?	87		75.7				15		13	13		11.3
Have other family members been neglected?	100		87				8		7	7		6.1
Disturbance of family leisure												
Have family recreational activities been stopped?	83		72.2				15		13	17	14.8	
Family members’ holidays and leaves are used to take care of the patient.	80		69.6				21		18.3	14	12.2	
Attention on children’s reduced and its effect on them?	87		75.7				17		14.8	11	9.6	
Due to the patient's illness, are any other leisure activities abandoned in the family?	88		76.5				15		13	12	10.4	
Disturbance of family interaction												
Does the family atmosphere get affected because of hospitalization?	30				26.1		64		55.7	21	18.3	
Do any arguments happen among the family members?	93				80.9		19		16.5	3	2.6	
Did relatives stop coming to the home?	102				88.7		8		7	5	4.3	
Does family members' relationship get affected because of hospitalization?	101				87.8		11		9.6	3	2.6	
Do the family members separate?	106				92.2		5		4.3	4	3.5	
Effects on the physical and mental health of other family members												
Does any of the family members physically get affected?		38			33		58		50.4	19	16.5	
Does any of the family members show adverse effects on their health?		42			36.5		55		47.8	18	15.7	
Does any of the family members asked help for their psychological problems?		27			23.5		58		50.4	30	26.1	
Does any other family members afflicted psychologically and expressing symptoms?		24			20.9		61		53	30	26.1	
Total burden score: (Mean ± SD =14.1 ± 7.3)												

Association between the socioeconomic burden of critically ill patients with their demographic variables

Table 4 presents the association between critically ill patients' socioeconomic burden and demographic variables. Patients aged less than 40 years (p-value = 0.018), those families who were fully dependent on the patient's income and occupation (p-value = 0.003) for their livelihood, and families with lower socioeconomic (p-value = 0.046) class status were significantly affected.

**Table 4:** Association between socioeconomic burden with socioeconomic variables of critically ill patients.

		Mean ± SD	F-value	P-value	Confidence Interval (95% CI)
Age group	≤40 (N=35)	16.3 ± 6.6	3.057	0.018	14.0-18.6
	41-50 (N=21)	14.7 ± 7.8			11.1-18.2
	51-60 (N=32)	14.4 ± 7.9			11.6-17.3
	>60 (N=27)	10.6 ± 5.8			8.3-12.9
Level of dependence	No dependency	10.9 ± 4.2	6.232	0.003	9.5-12.4
	Partial	14.5 ± 7.2			12.0 ± 17.0
	Full	16.4 ± 8.4			13.9-18.9
Socioeconomic class	Upper and Upper Middle	11.6 ± 6.4	3.170	0.046	8.0-15.2
	Lower Middle	12.3 ± 4.9			10.5-14.1
	Lower and Upper Lower	15.5 ± 8.0			13.6-17.4

## Discussion

The social and economic burden can be associated with any illness. Critical illness can affect any individual, including the most economically productive individuals in the family. Treatment of critical illness is associated with huge healthcare expenses to the patients and families that include both direct and indirect costs. Direct costs in the form of hospital admission, treatment, medications, transportation, cost of food and shelter to family members, and indirect costs such as missed school days or work and loss of work during the illness, hospitalization, loss of potential income for patient and caregiver and are often associated with morbidity and mortality.

Economic impact of critical illness on patients and families

This study was carried out in a government tertiary care hospital among critically ill patients and their family members. The cost of the treatment of the admitted patients was borne by the government for BPL patients and through government-funded health insurance schemes (GFHI) for eligible patients. The cost of treatment for other patients was borne by themselves on nominal charges. Other expenses for additional medications, transport, food, and shelter for family members were not covered under these schemes and were borne by patients and their families from their savings. The mean institution cost was INR 6404 (US$81.81), including the cost of investigations and treatment procedures not covered under these BPL and GFHI schemes. The results of a retrospective study done in the US show that 13.4% of hospital costs are accountable for intensive care [8]. The mean pharmacy cost was INR 38,169 (US$487.61), which includes the cost of medications used for the patients during the treatment and hospitalization that were unavailable or uncovered in the schemes. Medications were found to be an additional principal contributor to the direct cost during illness [9]. The ICUs are consuming a large share of hospital resources. Critically ill patients in the ICUs usually have longer lengths of stay and higher total costs when compared with other non-ICU patients [10]. In this study, 77% of the patients belonged to rural areas and traveled to urban areas for critical care. This cost of transportation also contributes to the economic burden on families. The transport cost must be decreased through the public transport system or by providing medical transport buses in rural places [11]. Most of the patients belonged to rural areas, and family members had to spend money for food and stay, increasing their expenses. The cost of treatment has a large variation in each component. The disproportion between hospital expenses and patients' income contributes to disastrous consequences for patients' families and forces them to get additional loans for treatment or lose hope of survival, leading to treatment termination. To cope with the unexpected financial loss of poor communities and meet the unmet expenses, the health schemes must include additional funds for these direct and indirect costs of critically ill patients.

Social impact of critical illness on patients and families

The mean age of the patients was 42 years. A total of 77% of patients belonged to the economically productive age group between 19 and 59 years. They are also the breadwinner on whom the survival and development of children and families depend. In about 49% of families, the patient himself was the head of the family. Around 48% of families had more than five dependent family members, which also increases the economic burden on the family. Loss of wages and productivity losses were measured as the lost man-days from productive activities because of illness and hospitalization. Also, those families solely depending on the patient's income and those aged less than 40 significantly reported a higher socioeconomic burden. The mean man-days lost was 17 days because of hospital visits before admission. The average man-days lost by the patient was 35 days during hospitalization, and the average man-days lost by the family members on account of care of the patients was 67 days. When the patient becomes ill and is hospitalized, the patient himself loses man-days of employment, and the family members, on account of attending to the patient, also suffer from a loss of man-days of employment which will eventually decrease the family's income and lead to the financial burden on the family. From these observations, it is clear that even though diagnosis and treatment are offered free of charge to patients attending government healthcare institutions, a significant financial loss is experienced by the patient's families. Also, the direct and indirect costs may vary based on the nature of the critical illness and the length of stay in the hospital or ICU. When the hospital stay increases, the risk of financial burden also increases for patients and their families. The potential risk of health-related work impairment due to post-critical illness also contributes to lowering the income and increases the economic burden on patient families. There is a greatest need and challenge to decrease the money spent out of their pockets during hospitalization with critical illness in ICUs. The government-funded insurance schemes must meet the patients' unmet financial needs, eventually decreasing the potential risk of poverty in the rural population during critical illness.

Limitations

In this study, we did not assess the direct costs spent by patients' families for diagnosis and treatment before hospitalization or ICU admission in government healthcare centers. Also, we did not assess the costs and financial loss that occurred after discharge. To generalize these findings, a similar study with a large sample size, including public and private healthcare centers, might assess the financial gap between healthcare's direct and indirect costs borne by the patients covered under government health schemes and other insurances.

## Conclusions

The study identifies that direct and indirect costs constitute the highest share of the total costs associated with critical illness for patients and their families. The socioeconomic effect of intensive care admissions extends beyond individuals or family members, especially in developing countries like India. Family members of critically ill patients admitted to the ICU experience significant problems in regard to psychological and social functioning. Further investigation is required to adopt effective policies to help the patients financially and positively impact family members during critical illness.
